# Topology of a G-quadruplex DNA formed by *C9orf72* hexanucleotide repeats associated with ALS and FTD

**DOI:** 10.1038/srep16673

**Published:** 2015-11-13

**Authors:** Bo Zhou, Changdong Liu, Yanyan Geng, Guang Zhu

**Affiliations:** 1 Division of Life Science, The Hong Kong University of Science and Technology, Clear Water Bay, Kowloon, Hong Kong SAR, PRC

## Abstract

Abnormal expansions of an intronic hexanucleotide GGGGCC (G4C2) repeat of the *C9orf72* gene are the most common genetic cause of amyotrophic lateral sclerosis (ALS) and frontotemporal dementia (FTD). Previous studies suggested that the *C9orf72* hexanucleotide repeat expansion (HRE), either as DNA or the transcribed RNA, can fold into G-quadruplexes with distinct structures. These structural polymorphisms lead to abortive transcripts and contribute to the pathogenesis of ALS and FTD. Using circular dichroism (CD) and nuclear magnetic resonance (NMR) spectroscopy, we analyzed the structures of *C9orf72* HRE DNA with various G4C2 repeats. They exhibited diverse G-quadruplex folds in potassium ions. Furthermore, we determined the topology of a G-quadruplex formed by d(G4C2)_4_. It favors a monomeric fold and forms a chair-type G-quadruplex with a four-layer antiparallel G-tetra core and three edgewise loops, which is distinct from known structures of chair-type G-quadruplexes. Our findings highlight the conformational heterogeneity of *C9orf72* HRE DNA, and may lay the necessary structural basis for designing small molecules for the modulation of ALS/FTD pathogenesis.

Amyotrophic lateral sclerosis (ALS) and frontotemporal dementia (FTD) are devastating neurological diseases. ALS is the most frequent motor neuron disease, caused by the loss of motor neurons in brain and spinal cord, resulting in progressive muscle weakness and paralysis, ultimately leading to death from respiratory failure^1^,^2^. FTD is the second most common form of dementia in individuals younger than 65 years^3^. It is characterized by changes in personality or language impairment, due to the progressive degeneration of the frontal and anterior temporal lobes of the brain^4^. ALS and FTD represent a clinicopathological spectrum of neurological diseases^5^, which are characterized by the cytoplasmic aggregates of a nuclear protein TAR DNA-binding protein (TDP-43)^6^,^7^.

Recently, large expansion of a GGGGCC (G4C2) hexanucleotide repeat in the first intron of the *C9orf72* gene has been demonstrated to cause ALS and FTD^8^,^9^,^10^. The healthy control population carries fewer than 25 repeats of this hexanucleotide^11^, whereas the FTD and ALS patients carry very large expansions ranging between 700 and 1600 repeats^8^,^9^. The accumulation of transcripts containing the G4C2 hexanucleotide repeat expansion (HRE) as nuclear RNA foci is detectable in patient cells^8^,^11^,^12^,^13^,^14^, which may sequester and inactivate RNA binding proteins^15^. Additionally, the transcribed expanded repeats can undergo repeat-associated non-ATG initiated translation resulting in accumulation of a series of dipeptide repeat proteins^16^,^17^,^18^.

As recently reviewed, both *C9orf72* HRE DNA and RNA may contribute to the pathogenesis of ALS/FTD diseases through mechanisms associated with their structure polymorphisms^19^. The *C9orf72* HRE DNA and RNA enable the formation of complex structures including G-quadruplexes^15^,^20^,^21^. G-rich DNA and RNA sequences can form stable four-stranded structures known as G-quadruplexes, in which four guanine bases are connected through Hoogsteen hydrogen bonding to form a square planar structure or guanine tetrad. Two or more guanine tetrads stack to form a G-quadruplex, which is stabilized by monovalent cations^22^. G-quadruplexes form intra-molecular or inter-molecular structures from one, two or four strands of nucleic acids^23^. Defined by the orientation of the strand loops, G-quadruplexes adopt parallel, antiparallel, or mixed topology^23^,^24^,^25^. Genome-wide search has identified 376,000 potential quadruplex sequences in the human genome^26^. Recent advances in *in vivo* detection of G-quadruplex by structure-specific antibody and structure-binding ligands provide convincing evidence for the *in vivo* existence of G-quadruplex structures at telomeres and in human chromosomes^27^. DNA G-quadruplexes are enriched at telomeres and are also found in promoter regions of a number of oncogenes^28^,^29^. Due to the functional relationship between telomeres, oncogenes and cancer, great efforts have been devoted to find potential ligands that target G-quadruplexes to be applied in anticancer therapies^30^,^31^.

Recent research has been mainly focused on the structures of *C9orf72* HRE RNA, which form highly thermodynamically stable parallel G-quadruplex. Depending on the repeat lengths, G4C2 RNA repeats can form unimolecular and multimolecular G-quadruplexes *in vitro*^21^. Nucleolin, the most abundant non-ribosomal proteins found in the nucleolus^32^, was observed in *C9orf72* HRE RNA pull-downs and shown to be mislocalized in the nucleoli of patients carrying *C9orf72* HRE^15^. Additionally, as was demonstrated *in vitro*, nucleolin preferentially recognized the RNA G-quadruplex, rather than RNA sense or antisense hairpins^15^. The *C9orf72* HRE DNA G-quadruplex has also been investigated in several studies. By circular dichroism (CD) spectroscopy, the *C9orf72* HRE DNA were shown to form parallel (e.g. d(G4C2)G4), antiparallel (e.g. d(G4C2)_4_) or mixed G-quadruplexes (e.g. d(G4C2)_3_ and d(G4C2)_5_)^15^,^33^.

Till now, no detailed structure information of the *C9orf72* HRE G-quadruplex has been obtained, which is required for better understanding the formation of *C9orf72* HRE G-quadruplexes and the molecular mechanism underlying *C9orf72* HRE-caused ALS/FTD. Here we investigated the structures of *C9orf72* HRE DNA G-quadruplexes by CD and NMR spectroscopy, and determined the topology of d(G4C2)_4_, which forms a chair-type G-quadruplex with a four-layer antiparallel G-tetra core and three edgewise loops.

## Results

### *C9orf72* HRE DNAs form G-quadruplex structures

**Figure 1:**
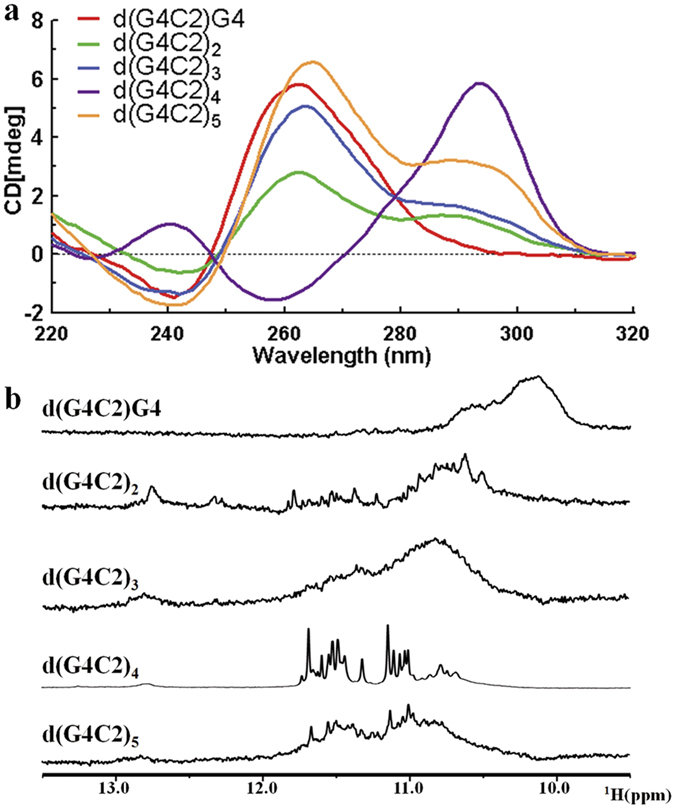
*C9orf72* HRE G4C2 DNAs form complex G-quadruplex structures. (**a**) CD spectra of d(G4C2)G4, d(G4C2)_2_, d(G4C2)_3_, d(G4C2)_4_ and d(G4C2)_5_. (**b**) The imino region of 1D ^1^ H spectra of various DNA samples.

In order to study the structural heterogeneity of *C9orf72* HRE DNA G-quadruplexes, we first screened DNA sequences with various d(G4C2) repeats for candidate DNAs that form G-quadruplexes and have sufficient quality for structural determination. We examined the secondary structures of these DNA samples using CD spectroscopy (Fig. 1a). The CD spectrum of d(G4C2)G4 in K+ solution displayed a negative and a positive peak at 240 nm and 260 nm, respectively, indicating a parallel G-quadruplex fold^23^. The d(G4C2)_4_ DNA in K+ solution showed a spectrum of predominantly antiparallel G-quadruplexes, which displayed a positive absorption peak at 295 nm and a negative peak at 260 nm^23^. The CD spectra of d(G4C2)_2_, d(G4C2)_3_ and d(G4C2)_5_ indicated a mixed form of parallel and anti-parallel G-quadruplex folds, which was consistent with the previous report^15^,^33^. The thermal stabilities of these five DNA samples were examined using CD melting experiments (Supplementary Fig. S1). The melting profiles of d(G4C2)G4, d(G4C2)_2_ and d(G4C2)_3_ showed no obvious transitions, suggesting that these DNA samples did not form stable G-quadruplex structures. In contrast, the melting curves of d(G4C2)_4_ and d(G4C2)_5_ exhibited apparent transitions, demonstrating that these two DNA samples formed stable G-quadruplex structures. The melting temperatures of d(G4C2)_4_ and d(G4C2)_5_ were around 90 °C and 75 °C, respectively, indicating that d(G4C2)_4_ formed the most stable G-quadruplex structure.

To determine the feasibility for pursuing further structural studies, 1D ^1^H NMR spectra of these DNA samples were also examined. The d(G4C2)G4, d(G4C2)_2_, d(G4C2)_3_ and d(G4C2)_5_ DNA samples appeared to form a mixture of multiple G-quadruplex conformations (Fig. 1b). Encouragingly, the 1D ^1^H NMR spectrum of the d(G4C2)_4_ DNA exhibited 16 well-resolved imino proton resonances at 10–12 ppm with narrow line widths (Fig. 1b), clearly demonstrating the formation of a predominant unimolecular G-quadruplex fold. The NMR melting experiment showed that the melting temperature of d(G4C2)_4_ was higher than 50 °C, which was consistent with the result obtained in the CD melting experiment (Supplementary Fig. S2).

To probe the molecular size of these DNAs, we performed the native polyacrylamide gel electrophoresis (PAGE) and employed DNA oligonucleotides dT21 and dT42 as size indicators (Supplementary Fig. S3). The d(G4C2)_4_ DNA migrated as a single band which was slower than dT21, but faster than dT42, suggesting that it existed as a homogeneous monomer. Other DNAs migrated as several bands indicating inhomogeneity in the sample composition and conformation. Because a homogeneous sample is required for high-resolution NMR structural study, we focused on d(G4C2)_4_ for further structural investigation.

### NMR assignment of guanines of d(G4C2)_4_ sequence

**Figure 2:**
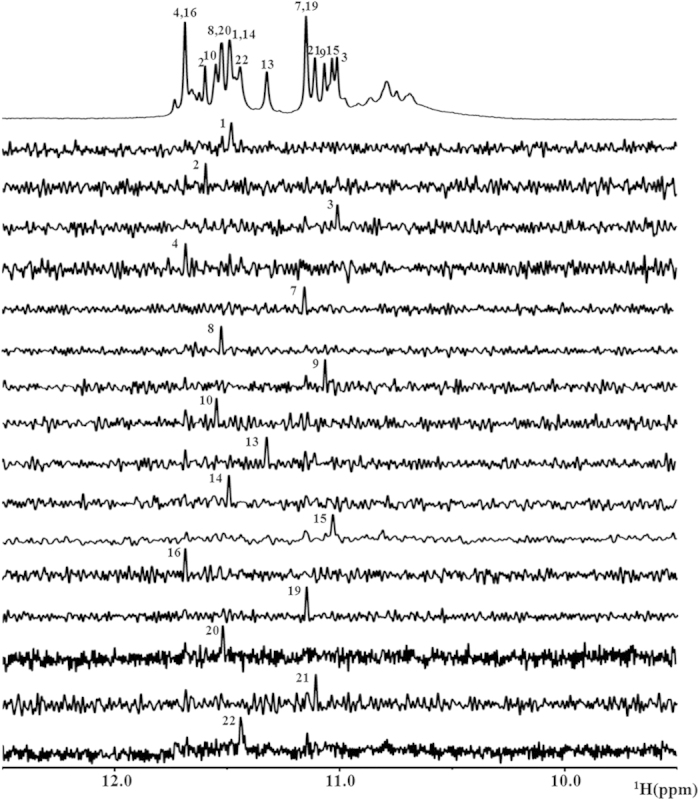
Imino proton (H1) assignments from ^15^N-filtered experiments. The imino region of 1D ^1^H NMR spectra with the assignment of guanine bases in d(G4C2)_4_ being indicated over the reference spectrum on top. Guanine imino protons were assigned using 1D ^15^N-filtered HSQC spectra of samples containing site-specific low-enrichment (2%) ^15^N-labelled oligonucleotides at the indicated positions.

The presence of 16 imino peaks in the 1D ^1^H spectrum of d(G4C2)_4_ in K+ solution indicates that all 16 guanines were involved in the intramolecular G-quadruplex formation and this G-quadruplex structure contained four layers of G-tetrads. In order to unambiguously assign guanine imino (H1) and aromatic (H8) proton resonances, low-enrichment ^15^N, ^13^C site-specific labeled oligonucleotides were synthesized^34^. The 1D ^15^N-filtered heteronuclear single quantum coherence (HSQC) spectra were recorded for these oligonucleotides (Fig. 2). One signal was observed in each of the spectra for the imino protons of the isotopically-labeled guanine bases, which demonstrated the single conformation of the d(G4C2)_4_ G-quadruplex in solution and was consistent with the PAGE result of d(G4C2)_4_ (Supplementary Fig. S3). Identified imino signals of 1D ^1^H-NMR spectra were shown in Fig. 2. The signals around 10–11 ppm are likely corresponding to imino protons of guanine residues involved in Hoogsteen hydrogen bonds in a minor conformation of d(G4C2)_4_.

The 2D ^13^C-^1^H HSQC spectra were also recorded for these oligonucleotides and aromatic proton assignments were obtained (Supplementary Fig. S4). These identified H1 and H8 signals were further confirmed by ^13^C-^1^H heteronuclear multiple bond correlation (HMBC) (Supplementary Fig. S5) and nuclear overhauser effect spectroscopy (NOESY) experiments^25^,^35^. The H1′ proton resonances of d(G4C2)_4_ were identified with the use of 2D NOESY spectra through correlations with H6/H8 protons of the same base. The 2D ^13^C-^1^H HSQC spectra for low-enrichment ^15^N, ^13^C site-specific labeled oligonucleotides also provided the H1′ proton resonances for cross-validation.

### d(G4C2)_4_ adopts an antiparallel chair type G4 topology

Based on the proton assignments obtained as described above, we analyzed the NOESY spectrum (75 ms mixing time) and COSY spectrum. The peak intensities of G1, G3, G7, G9, G13, G15, G19 and G21 were measured through non-linear fitting with the use of nmrDraw^36^ and gauged by the H5-H6 NOE peak intensities of cytosines to confirm the syn conformation of these guanosines (Supplementary Fig. S6). The remaining eight G-quadruplex guanines, namely G2, G4, G8, G10, G14, G16, G20 and G22, adopted the anti-conformation.

**Figure 3:**
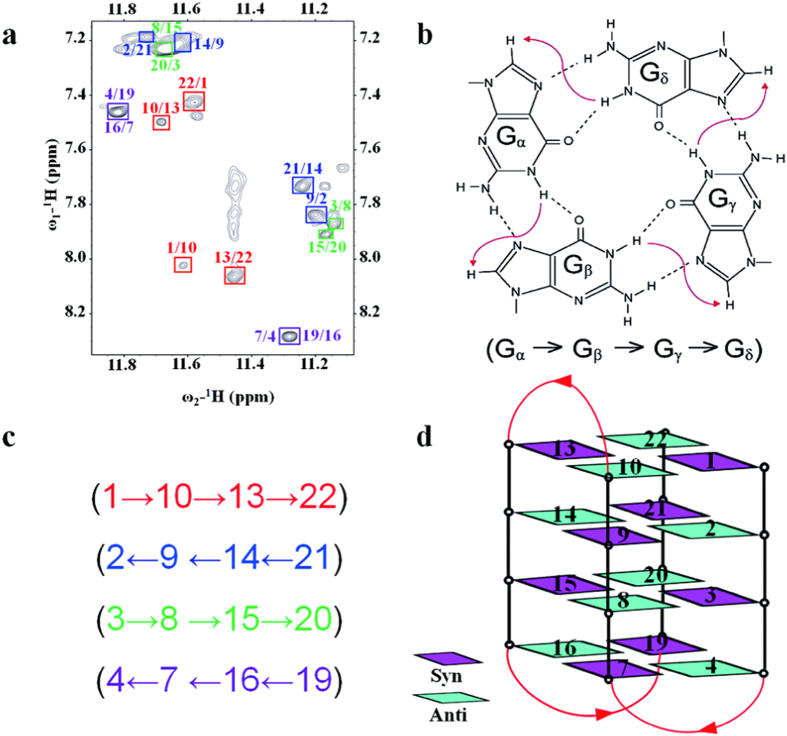
Topology determination for the *C9orf72* HRE DNA d(G4C2)_4_. (**a**) The NOESY spectrum (300 ms mixing time) showing H1–H8 connectivity. (**b**) Characteristic guanine H1–H8 NOE connectivity patterns around a Gα•Gβ•Gγ•Gδ tetrad as indicated by arrows. (**c**) Guanine H1–H8 NOE connectivities observed for the G1•G10•G13•G22, G2•G9•G14•G21, G3•G8•G15•G20 and G4•G7•G16•G19 tetrads. (**d**) Topology of d(G4C2)_4_. Anti guanines are colored in cyan, while syn guanines are colored in magenta. The backbones of the core and loops are colored in black and red, respectively.

In the current study, the characteristic synG(i)H1′/antiG(i + 1)H8 and synG(i)H8/antiG(i + 1)H1′ NOEs were observed, including synG1-antiG2, synG3-antiG4, synG7-antiG8, synG9-antiG10, synG13-antiG14, synG15-antiG16, synG19-antiG20 and synG21-antiG22 (Supplementary Fig. S7). The assignments of the imino H1 and base H8 protons of guanines in 2D NOESY spectrum (mixing time 300 ms) resulted in the direct determination of the folding topology of the G-quadruplex structure formed by d(G4C2)_4_ in K+ solution (Fig. 3a). In a G-tetrad plane with a Hoogsteen-type H-bond network, the imino proton H1 of a guanine was in close spatial vicinity to the base H8 of one of the adjacent guanines. Analysis of characteristic NOEs between H1 and H8 protons revealed the formation of an intramolecular G-quadruplex involving four G-tetrads: G1•G10•G13•G22, G2•G9•G14•G21, G3•G8•G15•G20 and G4•G7•G16•G19 (Fig. 3b–d). To resolve the ambiguity of the H8 chemical shifts of G4 and G16, and that of G7 and G19, we made a T substitution at C17 in the loop region, which was close to G16 and G19. The mutant C17T exhibited an excellent 1D ^1^H spectrum which closely resembled the wild type spectrum, indicating that the T substitution did not disrupt the wild type fold (Supplementary Fig. S8). Meanwhile, the peaks of G7 and G19 were clearly distinguished in the C17T spectrum. The 2D NOESY spectrum of C17T with 500 ms mixing time was also recorded. The characteristic NOE patterns of H1-H8 and H8-H1′ regions were consistent with the wild type while G4H8-H1′ and G16H8-H1′ were displayed as two individual peaks (Supplementary Fig. S9). Based on the characteristic NOEs between imino protons (G3H1-G7H1 and G15H1-G19H1) and the G(i)H2′/H2″-G(i + 1)H8 pattern, the G4H8 and G16H8 were unambiguously assigned (Supplementary Fig. S9). Due to the overlapped H8 chemical shifts of G7/G19 and G9/G21, and close H1′ chemical shifts of G8/G20 and G10/G22 in the H8-H1′ region of NOESY spectrum, synG(i)/antiG(i + 1) connectivities of synG7-antiG8, synG9-antiG10, synG19-antiG20 and synG21-antiG22 were further verified through the G(i)H2′/H2″-G(i + 1)H8 pattern based on the NOESY spectrum of C17T (Supplementary Fig. S9). Combined with the characteristic NOEs between H1 and H8 protons of C17T, we determined that the hydrogen-bond directionalities of the four G-tetrads were clockwise, anti-clockwise, clockwise and anti-clockwise, respectively. The glycosidic conformations of guanines around the tetrads were syn•anti•syn•anti. The G-tetrad core was of the antiparallel-type, in which each G-tetrad was oriented in opposite direction with respect to its neighboring ones (Fig. 3d).

## Discussion

Recent studies have shown that the *C9orf72* HRE DNA can form G-quadruplex structures, which was demonstrated mainly by CD spectroscopy^15^,^33^. *C9orf72* HRE DNAs with different repeat lengths adopted parallel, anti-parallel or mixed conformation. Sket *et al.* also obtained the 1D ^1^H NMR spectrum of the *C9orf72* HRE DNA, which indicated a G-quadruplex fold, but they failed to obtain homogeneous samples for 3D structure determination^33^.

In this study, we also employed CD and NMR spectroscopy to investigate the G-quadruplex formation by *C9orf72* HRE DNA. First, *C9orf72* HRE DNAs with various repeat lengths were screened for homogeneous samples that allow for structural determination by NMR. After the screening, d(G4C2)_4_ that adopted a single conformation was further pursued for structural investigations. By a combination of NMR approaches, we determined the topology of d(G4C2)_4_, which represents an antiparallel conformation adopted by the *C9orf72* HRE DNA. This DNA forms a chair-type G-quadruplex with a four-layer antiparallel G-tetra core and three edgewise loops. Although the chair-type topology for G-quadruplexes has been identified for a *Bombyx mori* telomeric DNA^37^ and a thrombin aptamer DNA^38^, the loop arrangements of these DNAs and d(G4C2)_4_ differ from each other in the length and sequence. The G-quadruplex of the *Bombyx mori* telomeric sequence, d[TAGG(TTAGG)_3_], contains a two-layer antipaprallel G-tetrad core and three edgewise loops, in which one loop (TTA) spans across a narrow groove and the other two loops (TTA/TTA) span wide grooves. The G-quadruplex of the thrombin aptamer DNA, d(GGTTGGTGTGGTTGG), also contains a two-layer antipaprallel G-tetrad core and three edgewise loops, in which one loop (TGT) spans a wide groove and the two remaining loops (TT/TT) span narrow grooves. In contrast, in our reported topology of d(G4C2)_4_, three edgewise loops are all composed of CC, which span both wide and narrow grooves.

*C9orf72* HRE DNA/RNA can fold into G-quadruplex structures and form transcriptionally induced R-loops^15^,^21^,^33^. These structural polymorphisms lead to abortive transcripts, which may act as fundamental determinants of the pathogenesis of *C9orf72* HRE-linked ALS/FTD. Therefore, detailed structure information of *C9orf72* HRE is in high demand for understanding their structural diversity and the molecular mechanism underlying *C9orf72* HRE-caused pathologies. Here we studied the structures of *C9orf72* HRE DNA with short repeat lengths, and demonstrated the structural variations of these G-quadruplex DNAs *in vitro*. The long expanded DNA repeats remain to be investigated for the structural features of these nucleic acids *in vivo*. Additionally, while d(G4C2)_4_ exhibited an antiparallel conformation, G4C2 HRE DNAs of other repeat lengths were found to adopt parallel or mixed conformation. Due to the inhomogeneity of the DNA samples, they were not applicable for further NMR investigations. Although not a focus in this study, some modification tools, like 8-bromo guanine substitution, may be used to improve the homogeneity of the samples to facilitate 3D structure determination of the parallel conformation adopted by the G4C2 HRE DNA.

Various small molecules have been employed to target G-quadruplex-forming sequences, to modulate telomere maintenance or gene activity^31^,^39^. As multimerization of *C9orf72* HRE G-quadruplexes may exacerbate the formation of RNA foci and cause neuron-toxicity in ALS/FTD^15^, disruption of these foci by targeting *C9orf72* HRE G-quadruplexes may have beneficial outcomes in the treatment of these diseases. Therefore, this study may also build the necessary basis for designing small molecules which can therapeutically target *C9orf72* HRE to modulate ALS/FTD pathogenesis.

## Methods

### Sample preparation

Unlabeled and site-specific low-enrichment (2% ^15^N labeled or ^15^N, ^13^C-labeled) DNA oligonucleotides were chemically synthesized and HPLC purified (Takara, China). DNA was annealed at the 0.1 mM single stranded concentration by heating to 95 °C for 15 min, followed with slow cooling to room temperature overnight in the annealing buffer containing 70 mM KCl and 20 mM potassium phosphate (pH 7.0). The NMR sample was concentrated by ultrafiltration with the use of a Centricon 3kD (Millipore, MA) in 20 mM potassium phosphate buffer (pH 7.0) and 70 mM KCl. The final concentration of the DNA sample was 2.5 mM.

### Circular dichroism

Circular dichroism spectra were recorded at 25 °C on a JASCO-815 CD spectropolarimeter using a 1 mm path length quartz cuvette with a reaction volume of 200 μl. DNA concentration was 20 μM. The DNA oligonucleotides were prepared in a 20 mM potassium phosphate buffer (pH 7.0) containing 70 mM KCl. The CD melting experiments were performed by measuring the absorbance at a single wavelength (260 nm for parallel G-quadruplexes and 295 nm for antiparallel G-quadruplexes) with a temperature range of 25 °C to 95 °C.

### Polyacrylamide gel electrophoresis (PAGE)

Non-denaturing PAGE were carried out on a 18% polyacrylamide gel (acrylamide:bis-acrylamide 29:1), supplemented with 20 mM KCl in the gel and running buffer (0.5X TBE). The samples were prepared at a strand concentration of 100 μM. Bands were revealed by red-safe staining.

### NMR spectroscopy

Experiments were performed on 500 MHz, 750 MHz and 800 MHz Varian spectrometers. Resonances were assigned unambiguously using samples with site-specific low-enrichment ^15^N or ^15^N, ^13^C labeling and through-bond correlations at natural abundance. Standard 2D NMR experimental spectra including NOESY, TOCSY and COSY, were collected at 25 °C to obtain the complete proton resonance assignments. The NMR experiments for samples in water solution were performed with Watergate or Jump-and-Return water suppression techniques. The variable temperature spectra were recorded on 500 MHz Varian spectrometer from 5 °C to 50 °C. All spectra were processed by the program NMRPipe. Spectral assignments were also carried out and supported by COSY, TOCSY and NOESY spectra. Peak assignments were achieved using the software Sparky.

## Additional Information

**How to cite this article**: Zhou, B. *et al.* Topology of a G-quadruplex DNA formed by *C9orf72* hexanucleotide repeats associated with ALS and FTD. *Sci. Rep.* **5**, 16673; doi: 10.1038/srep16673 (2015).

## Supplementary Material

Supplementary Information
