# Supplementary material for: Topology of a G-quadruplex DNA formed by C9orf72 hexanucleotide repeats associated with ALS and FTD
Source: Sci Rep. 2015 Nov 13;5:16673. doi: 10.1038/srep16673 (PMC4643247; doi:10.1038/srep16673)
Supplement: Supplementary Information [file srep16673-s1.pdf]

Topology of a G-quadruplex DNA formed by *C9orf72* hexanucleotide repeats associated with ALS and FTD

Bo Zhou, Changdong Liu, Yanyan Geng & Guang Zhu

Supplementary information

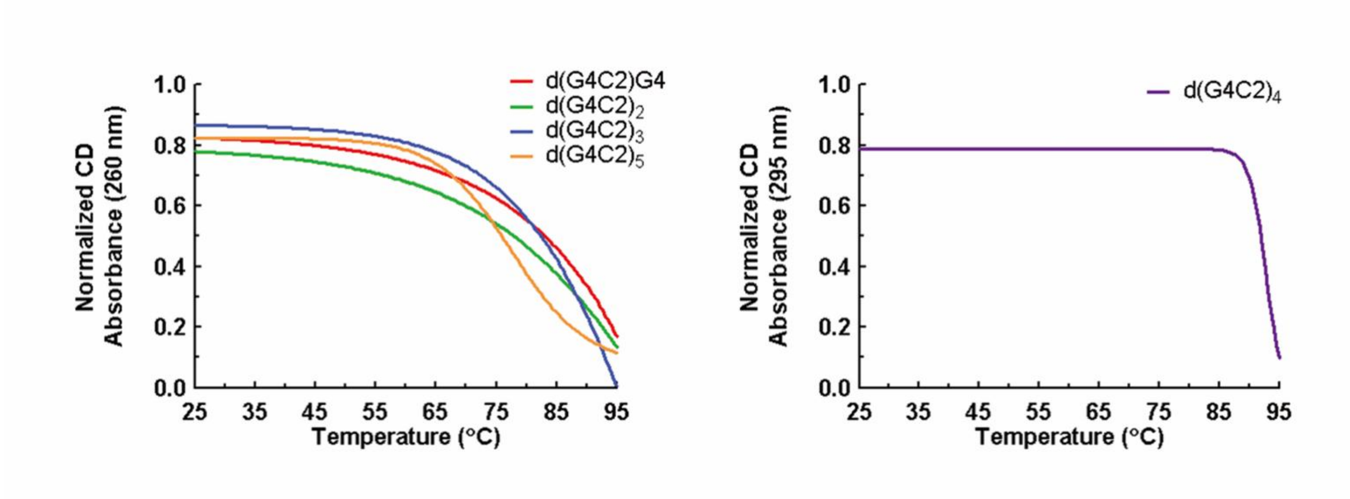

Supplementary Figure S1. CD melting curves of d(G4C2)G4, d(G4C2)$_2$, d(G4C2)$_3$, d(G4C2)$_4$ and d(G4C2)$_5$. CD absorbances were normalized by using the equation (Abs_t–min)/(max – min), in which Abs_t is the absorbance at a given temperature, max is the maximum absorbance at 260 nm for parallel G-quadruplexes and at 295 nm for antiparallel G-quadruplexes, and min is the minimum value. Data were fit by the Boltzmann sigmoid equation (Prism).

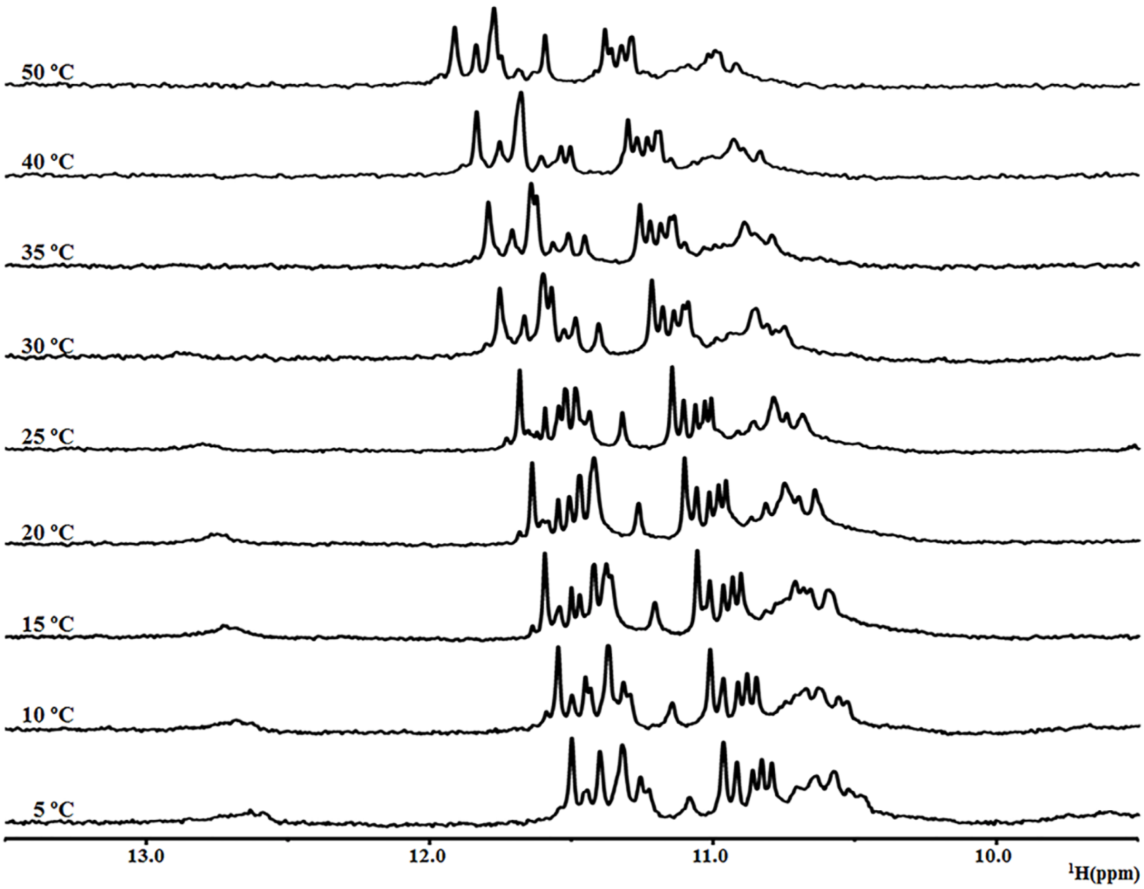

Supplementary Figure S2. NMR melting experiment for d(G4C2)$_4$. Imino proton regions of variable temperature 1D ^{1}H NMR spectra of d(G4C2)$_4$.

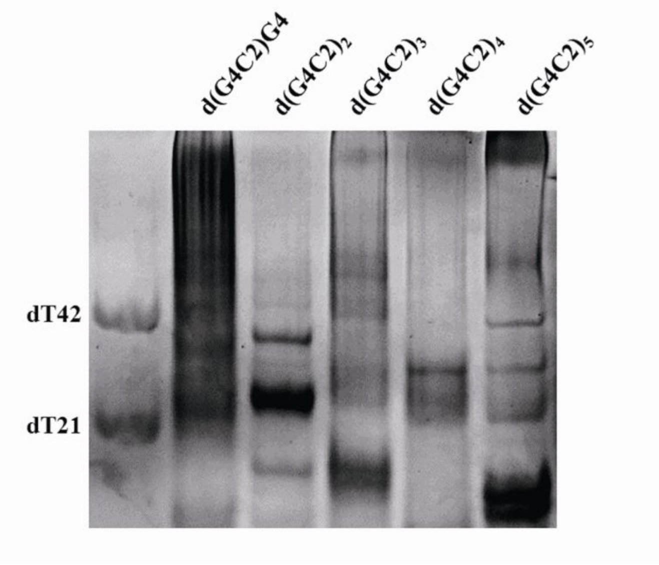

Supplementary Figure S3. Gel electrophoresis of G4C2 DNAs monitored by staining method. Non-denaturing 18% PAGE for *C9orf72* HRE G4C2 DNAs at 100 μM concentration.

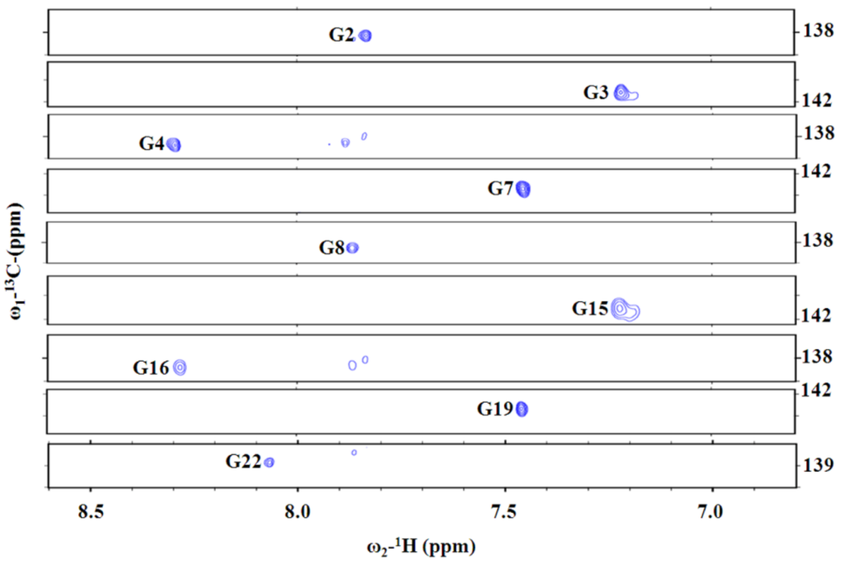

Supplementary Figure S4. Aromatic proton (H8) assignment from ^{13}C-^{1}H HSQC experiments. The expanded H8-C8 regions of ^{13}C-^{1}H HSQC spectra for G2, G3, G4, G7, G8, G15, G16, G19 and G22. In each spectrum the low-enrichment ^{15}N, ^{13}C labeled guanines at specific site is indicated. The ^{13}C-^{1}H HSQC spectra for G1, G9, G10, G13, G14, G20 and G21 that gave low quality were not shown and they were assigned through ^{13}C-^{1}H HMBC.

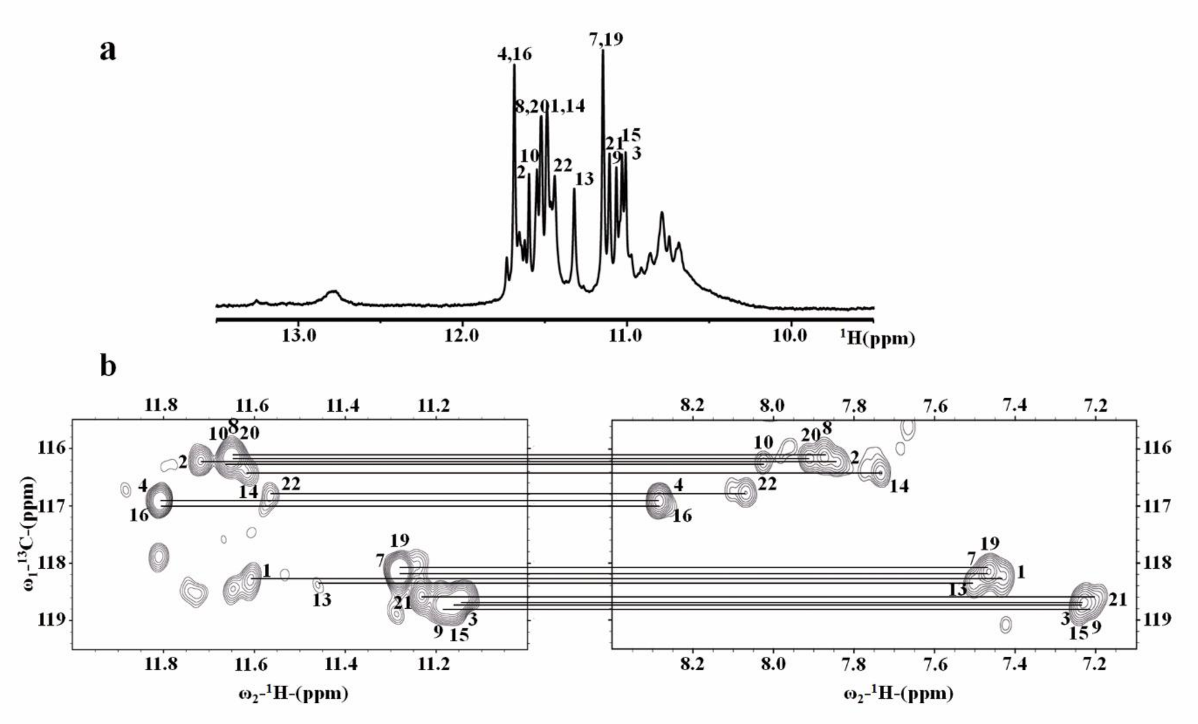

Supplementary Figure S5. The H1 and H8 regions of 2D ^{13}C-^{1}H HMBC spectrum. (a) The imino region of 1D ^{1}H-NMR spectrum with the assignment of guanine bases in d(G4C2)$_4$ being indicated. (b) The H1 and H8 regions of 2D ^{13}C-^{1}H HMBC spectrum showing the correlation between H1 and H8 protons within guanine bases.

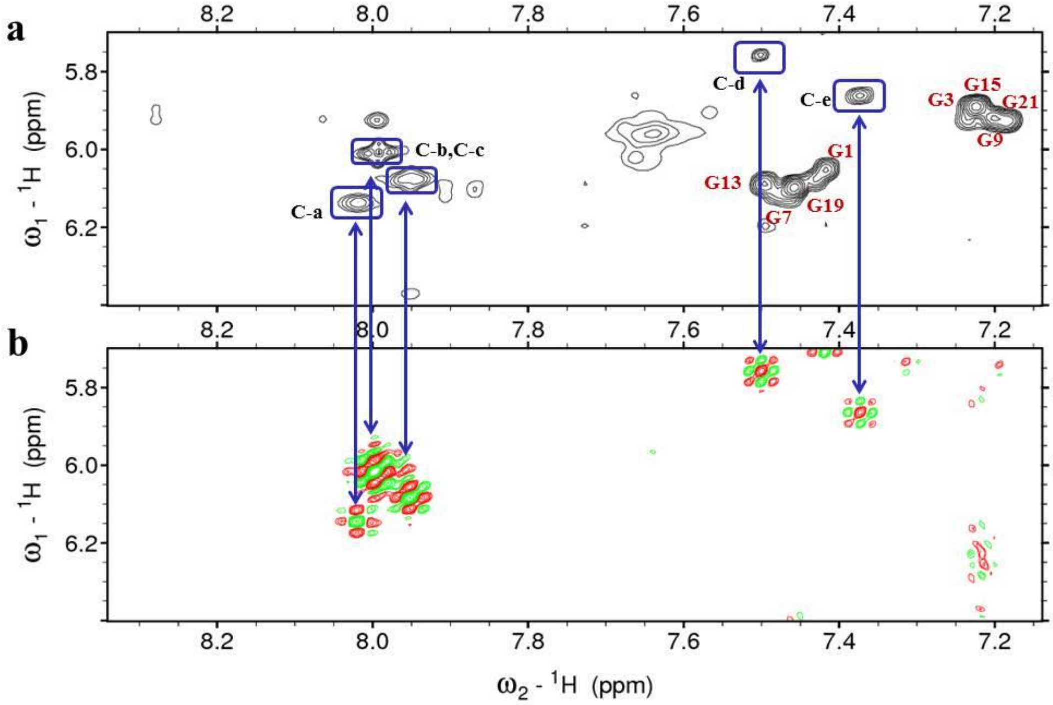

Supplementary Figure S6. Determination of guanines in *syn* conformation. (a) Strong intra residue guanosine H8-H1' cross-peaks (syn glycosidic bonds) are labeled in the NOESY spectrum (75 ms mixing time). The remaining strong peaks observed in this spectrum are from cytosines (named as C-a, C-b, C-c, C-d and C-e),which are identified through the COSY spectrum. The peak intensities of G1, G3, G7, G9, G13, G15, G19 and G21 are measured through non-linear fitting with the use of nmrDraw and gauged by the H5-H6 NOE peak intensities of cytosines to further confirm the syn conformation of these guanosines. The list of the peak intensity:

**Peak number**	**C-a**	**C-b**	**C-c**	**C-d**	**C-e**	**G1**	**G3**	**G7**	**G9**	**G13**	**G15**	**G19**	**G21**
**Peak Intensity (e+06)**	**0.7533**	**0.3598**	**0.4655**	**0.7345**	**0.8983**	**1.770**	**4.053**	**4.049**	**2.384**	**1.621**	**4.053**	**4.049**	**2.245**

(b) The H8/H6-H1' region of the COSY spectrum. The H5-H6 cross peaks of cytosines in NOESY and COSY spectra are indicated by blue double headed arrows.

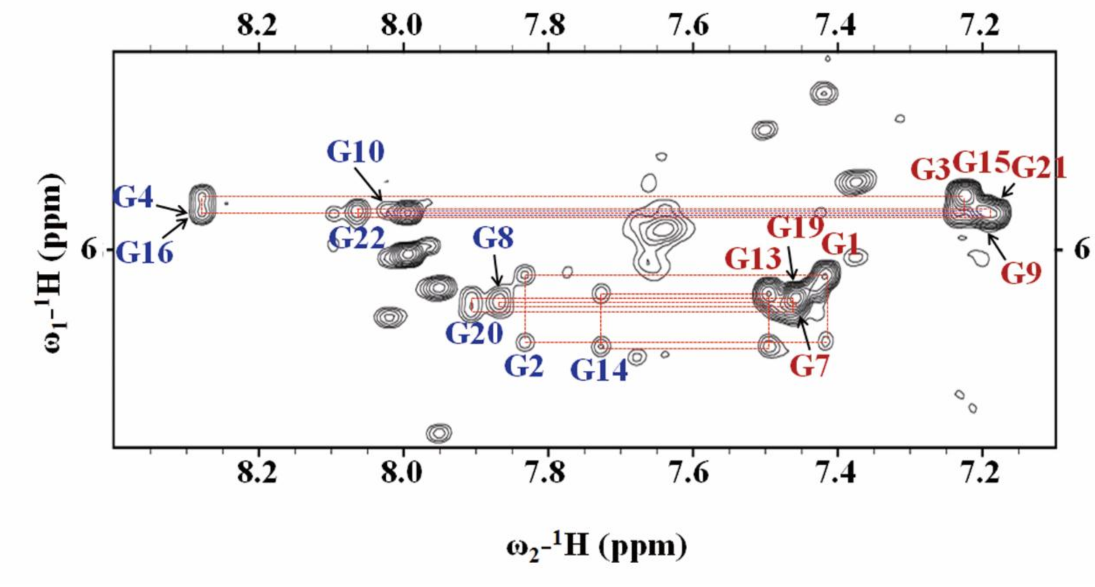

Supplementary Figure S7. NOESY spectrum (500 ms mixing time) showing the H8-H1' connectivity of d(G4C2)$_4$. The characteristic *syn*G(i)H1'/*anti*G(i+1)H8 and *syn*G(i)H8/*anti*G(i+1)H1' NOEs were observed, including *syn*G1-*anti*G2, *syn*G3-*anti*G4, *syn*G7-*anti*G8, *syn*G9-*anti*G10, *syn*G13-*anti*G14, *syn*G15-*anti*G16, *syn*G19-*anti*G20 and *syn*G21-*anti*G22.

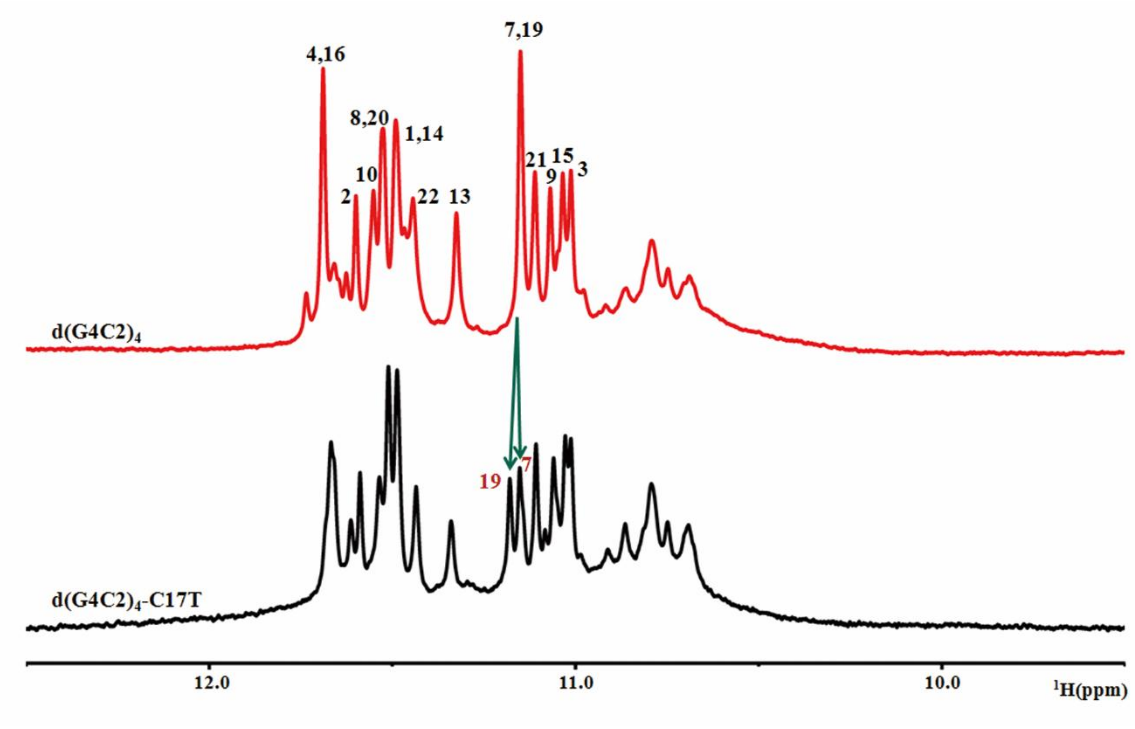

Supplementary Figure S8. The imino region of 1D proton spectra of the wild type d(G4C2)$_4$ (top: red) and the mutant d(G4C2)$_4$-C17T (bottom: black). The peak containing two bases 7 and 19 in the wild type d(G4C2)$_4$ splitted into two peaks in the mutant d(G4C2)$_4$-C17T.

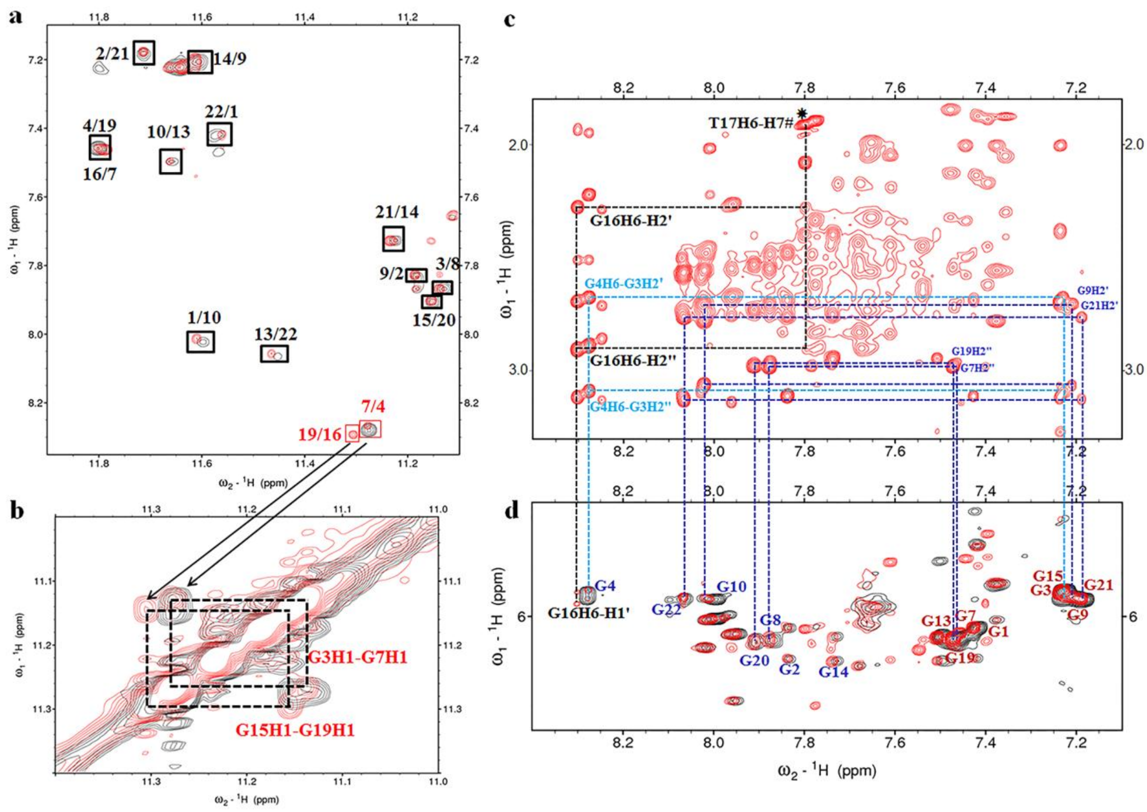

Supplementary Figure S9. The ^{1}H-^{1}H NOESY spectra of the wild type d(G4C2)$_4$ (black) and the mutant d(G4C2)$_4$-C17T (red) with a 500 ms mixing time. (a) The expanded ^{1}H-^{1}H NOESY spectrum showing the H1-H8 connectivity of the wild type d(G4C2)$_4$ (black) is overlaid with that of the mutant d(G4C2)$_4$-C17T (red). (b) The expanded H1-H1 region of the ^{1}H-^{1}H NOESY spectrum of the wild type d(G4C2)$_4$ (black) is overlaid with that of the mutant d(G4C2)$_4$-C17T (red). (c) The expanded H8/H6-H2'/H2" region of the ^{1}H-^{1}H NOESY spectrum of the mutant d(G4C2)$_4$-C17T (red). (d) The expanded 1H-1H NOESY spectrum correlating base H8 and sugar H1' protons of the wild type d(G4C2)$_4$ (black) is overlaid with that of the mutant d(G4C2)$_4$-C17T (red). The G(i)H2'/H2"-G(i+1)H8 connectivity is plotted for G3-G4,G7-G8,G9-G10,G19-G20 and G21-G22 in (c) and (d).
